# Successful Management of Multifactorial Colitis in a Recipient of Hematopoietic Stem Cell Transplant: A Case Report

**DOI:** 10.4137/ccrep.s838

**Published:** 2008-07-29

**Authors:** Khalid A. Al-Anazi, Asma M. Al-Jasser, Amal Abdulwahab, Entezam Sahovic, Hadeel Almana, Mohammed A. Al Fadda

**Affiliations:** 1 Section of Adult Hematology and Hematopoietic Stem Cell Transplant, King Faisal Cancer Centre, King Faisal Specialist Hospital and Research Centre, P.O. Box: 3354, Riyadh 11211, Saudi Arabia.; 2 Departments of Medicine and Pathology, King Faisal Specialist Hospital and Research Centre, P.O. Box: 3354, Riyadh 11211, Saudi Arabia.; 3 Section of Microbiology, Department of Pathology, Armed Forces Hospital, P.O. Box: 7897, Riyadh 11159, Saudi Arabia.

**Keywords:** acute lymphoblatic leukemia, graft versus host disease, hematopoietic stem cell transplant, cytomegalovirus

## Abstract

Recipients of allogeneic hematopoietic stem cell transplant can develop life-threatening complications at any time following their transplants. These complications require repeated clinical assessment, appropriate and thorough screening as well as a comprehensive management approach.

We report a young adult male who received a sibling allograft in the second complete remission of his acute lymphoblastic leukemia at King Faisal Specialist Hospital and Research Centre in Riyadh. The patient developed severe colitis which was caused by: acute exacerbation of chronic graft versus host disease of the lower gastrointestinal tract, cytomegalovirus disease of the colon and a superadded *Salmonella* infection caused by food poisoning. The multifactorial colitis was properly investigated and successfully managed. To our knowledge, this is the first case of multifactorial colitis in a recipient of hematopoietic stem cell transplant.

## Introduction

Young adult acute lymphoblastic leukemia (ALL) patients between 15 and 20 years of age can be treated with either adult or pediatric ALL protocols (Vitale et al. 2006). Adolescents treated in adult trials appear to have a significantly higher risk of treatment failure resulting mostly in refractory leukemia (Vitale et al. 2006). The outcome of these patients is markedly improved if the intensive pediatric protocols rather than the less intensive adult ALL protocols are used in their treatment (Machman, 2005).

A proportion of patients with relapsed leukemia can be cured with allogeneic hematopoietic stem cell transplant (HSCT). However, selection of patients with <30% blasts, identification of young human leucocyte antigen (HLA)-matched donors and use of total body irradiation (TBI) may significantly improve the outcome in this group of patients (Bacigalupo et al. 2007). Intense pre-HSCT conditioning regimens, e.g. those containing TBI, are usually associated with a lower rate of leukemic relapse, but an increase in the rate of transplant-related mortality (Clift et al. 1991).

## Case Report

A 17 year old Saudi male was diagnosed to have Pre–B ALL in March 2002 at King Fahad National Guard Hospital in Riyadh. He presented with fever, mucosal bleeding, generalized external lymphadenopathy, splenomegaly, thrombocytopenia, normal WBC count and Hb level, blasts on the blood film and a heavy infiltration of the bone marrow with blast cells in addition to complex cytogenetic abnormalities. He received an induction course of chemotherapy composed of cyclophosphomide, daunorubicin, vincristine, L-asparaginase, prednisone and intrathecal methotrexate, following which the first complete remission (CR) of his leukemia was achieved. Thereafter, the patient was given consolidation and maintenance courses of chemotherapy till March 2004. In February 2005, he had relapse of his ALL with almost the same clinical and laboratory abnormalities. However, he achieved a second CR following a salvage course of chemotherapy composed of high dose cytosine arabinoside and idarubicin. After identifying a healthy and an HLA-identical sibling donor, the patient was transferred to King Faisal Specialist Hospital and Research Centre (KFSH&RC) in Riyadh for an allogeneic HSCT. On admission to the HSCT unit on 18/4/05, he was asymptomatic and his physical examination revealed no abnormality. Complete blood count (CBC) showed WBC: 3.72 × 10^9^/L, Hb: 136 g/L and PLT: 256 × 10^9^/L. Blood film showed no blast cells and pre-HSCT bone marrow biopsy showed no evidence of leukemia. The patient received a conditioning protocol composed of cyclophosphamide and TBI. He was given acyclovir, trimethoprim-sulphamethoxa-zole (TMP/SMZ) and fluconazole as infection prophylaxis as well as cyclosporine and methotrexate as graft versus host disease (GVHD) prophylaxis. On 25/4/2005, the patient received his allograft without any complications. In the early post-HSCT period, he developed grade I mucositis treated with IV morphine infusion and two febrile neutropenic episodes treated empirically with tazobactampiperacillin, gentamicin and vancomycin. He engrafted his leucocytes on day +20 and his platelets on day +12 HSCT. On day +28 HSCT, the patient developed acute GVHD of the skin, which was treated with IV 6-methylpednisolone then oral prednisone. After controlling the skin GVHD, he was sent home on cyclosporine, zantac and prophylactic antimicrobials in addition to the tapered doses of prednisone.

**Figure 1:**
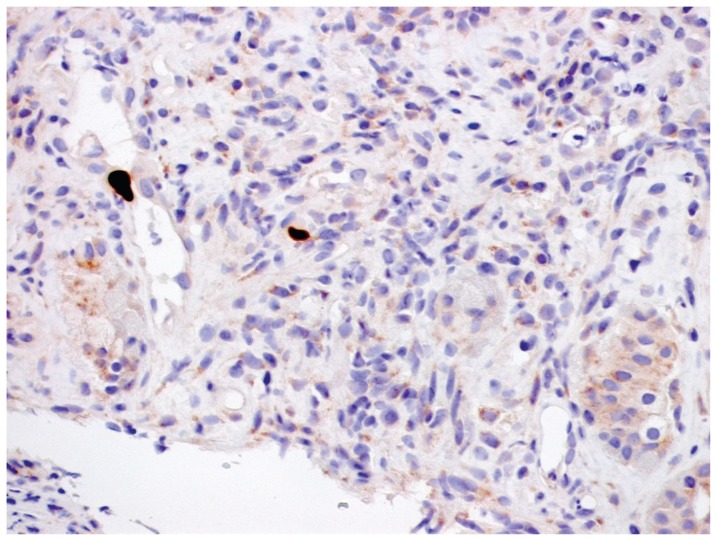
It shows changes consistent with severe colitis due to both graft versus host disease and cytomegalovirus infection.

On day +135 HSCT, the patient was readmitted with an extensive chronic GVHD involving skin, mouth, colon and liver. He presented with fever, abdominal pain and diarrhea for one week and he gave history of taking food from a restaurant. Physical examination revealed an unwell young male with generalized pigmentation, jaundice and lichenoid oral eruptions but no leg edema or external lymphadenopathy. The chest was clear, the cardiovascular and the neurological examinations were normal but examination of the abdomen revealed diffuse tenderness, positive bowel sounds and no hepatosplenomegaly. The CBC showed: WBC: 6.3 × 10^9^/L, Hb: 160 g/L and PLT: 271 ×10^9^/L. The renal function tests were normal. The hepatic profile revealed: serum bilirubin: 96 μmol/L, albumin: 35 g/L, ALT: 558 U/L, AST: 209 U/L and alkaline phosphate: 404 U/L. Colonoscopy was done and it showed grade I GVHD of the colon. The patient was kept on bowel rest and he was commenced on: intravenous (IV) fluids, IV methylprednisolone 1mg/kg twice daily, IV zantac 50 mg thrice daily and IV immunoglobulin 0.4 g/kg weekly in addition to prophylactic acyclovir and TMP/SMZ. The stool cultures grew *Salmonella* species so the patient received IV ceftriaxone 2 grams daily for 10 days. As diarrhea continued, the patient was commenced on: mycophenolate mofetil (MMF) 500 mg twice daily, ursodeoxycholic acid 300 mg orally twice daily, IV omeprazole and octreotide 50 μg IV thrice daily. On day +145 HSCT, he was started on IV ganciclovir: 5 mg/kg twice daily as cytomegalovirus (CMV) antigen test became positive. A repeat colonoscopy showed severe colitis with grade IV colonic GVHD (Fig. 1). Later on, the patient continued to have low grade pyrexia, abdominal pain and diarrhea so he was commenced on total parenteral nutrition and rabbit anti-thymocyte globulin 1.5 mg/kg on alternative days for a total of 3 doses and cyclosporine was replaced by tacrolimus. On day +152 HSCT, the patient was still having positive CMV antigenemia and the previously taken colonic biopsy became positive for CMV (Fig. 1) so IV ganciclovir was replaced by IV foscarnet 60 mg/kg thrice daily. Few days later, the bloody diarrhea and the abdominal pains decreased significantly, then the patient continued to improve clinically. On day +177 HSCT, the patient was having no more diarrhea or abdominal pain, so he was shifted to oral prednisone 1.5 mg/kg/day for 3 days and a maintenance dose of IV foscarnet 100 mg/kg/day. Four days later, the patient was asymptomatic and his physical examination showed hyperpigmentation but no jaundice or abdominal tenderness. The blood counts showed: WBC: 2.02 × 10^9^/L, Hb: 10/g/L and PLT: 73 × 10^9^/L. The renal function tests were all normal. The hepatic profile showed: bilirubin 82 μmol/L, ALT: 100 U/L and AST 31 U/L. The patient was sent home on: foscarnet 500 mg IV once daily for one more week, tacrolimus 5 mg orally twice daily, MMF 1 gram twice daily, prednisone 50 mg/day and ursodeoxycholic acid 150 mg daily. Thereafter, the patient had a regular follow up at the HSCT outpatient clinic and he sustained his clinical and laboratory improvement which allowed gradual tapering of his immunosuppressive therapy.

## Discussion

Infections represent one of the most important complications of allogeneic HSCT, with a negative impact on survival and on the quality of life (Wingard, 1999). In a retrospective study on infectious complications in allogeneic transplant recipients randomised to receive bone marrow or peripheral blood stem cells (PBSCs), a multivariate analysis of factors associated with infection episodes was performed. The duration of corticosteroid use was the single variable significantly associated with more episodes of infection (Nucci et al. 2003). Corticosteroids depress a number of host defences including: chemotaxis of neutrophils, macrophage function and cellular immunity (Nucci et al. 2003).

CMV infection remains a serious complication of allogeneic HSCT (Ljungman et al. 2004). The virus can infect cells of the 2 functional compartments of the bone marrow: the hematopoietic and the stromal compartments (Steffens et al. 1998). CMV inhibits the engraftment of the transplanted hematopoietic cells, thus ultimately leading to a complete graft failure (Steffens et al. 1998). During the last 15 years, several techniques have been developed that allow rapid and sensitive diagnosis of CMV infection (Ljungman et al. 2004). Allogeneic HSCT recipients should be monitored for CMV in the peripheral blood at least weekly by either the CMV antigenemia assay or a technique for the detection of CMV DNA or RNA for a total period of at least 100 days. In allogeneic HSCT recipients, acyclovir or valaciclovir may be used as prophylaxis against CMV and either IV ganciclovir or foscarnet can be used for first line pre-emptive antiviral therapy (Ljungman et al. 2004).

CMV disease remains a serious infectious complication that causes morbidity and mortality in recipients of allogeneic HSCT (Meyers et al. 1990). In allogeneic HSCT patients, CMV-gastrointestinal tract (GIT) disease can occur even in those having strict CMV surveillance using CMV antigenemia or real-time polymerase chain reaction (PCR) and receiving pre-emptive antiviral therapy (Mori et al. 2004). Studies have shown that CMV disease can occur at anytime after HSCT from the early neutropenic period to several years after transplantation. Currently, at least 50% of cases of CMVdiseasedeveloplaterthan100days after transplantation (Boeckh et al. 2003). The diagnosis of CMV disease must be based on consistent symptomatology together with the detection of CMV in the appropriate specimen from the involved tissue (Ljungman et al. 2004). For types of CMV disease other than CMV pneumonitis, either IV ganciclovir or foscarnet without the addition of immunoglobulin is recommended (Ljungman et al. 2004). However, either cedofovir or the combination of IV gancilovir and foscarnet, each given in full dosage, may be used as second-line treatment for CMV disease. There is no standard therapy duration for CMV disease, but a commonly used schedule is 21–28 days of induction therapy followed by maintenance treatment for 4 weeks (Ljungman et al. 2004).

Resistance of CMV to antiviral agents is a well documented complication of long-term antiviral therapy (Ljungman et al. 2004; Erice, 1999). Current data suggest that the incidence of infections caused by drug-resistant CMV in HSCT recipients and solid organ transplant patients is on the low side (Erice, 1999). There are 2 types of resistance: clinical and viral. Clinical resistance depends on host factors, while viral resistance is due to mutations in the viral genome. Rising CMV antigenemia or DNA levels or progress of CMV disease symptoms may indicate clinical or viral reistance (Ljungman et al. 2004). Where possible, resistance testing should be performed to allow selection of the correct second line antiviral therapy. If test results are delayed, then a change of treatment for a patient with rising viral load or worsening disease due lack of adequate treatment may be justified (Ljungman et al. 2004).

Despite significant progress in the past 20 years, GVHD remains a significant cause of morbidity and mortality after allogeneic HSCT. Current approaches for prevention and treatment of GVHD involve direct blockade of T-cell function by various pharmacological agents. Most early trials have shown that T-cell depletion can substantially limit acute and chronic GVHD (Ho & Soiffer, 2001). GVHD can be classified into acute or chronic based on timing of onset and clinical manifestations (Nash et al. 2000). Acute GVHD usually develops within the first 2 months of HSCT and affects skin, GIT and liver (Nash et al. 2000). When pharmacological immunosuppression is used as GHVD prophylaxis after myeloablative HSCT, moderate to severe (grades II to IV) acute GVHD occurs in 25%–60% of matched related donor transplant recipients and upto 45%–70% of unrelated donor transplant recipients (Nash et al. 2000). Development of grades II to IV acute GHVD is associated with decreased survival in patients after allogeneic HSCT (Nash et al. 1992). Treatment of acute GVHD is difficult because steroids and other immunosuppressive agents increase the likelihood of lethal infections, whether or not GVHD has been controlled (Antin et al. 2004). A recent study showed that five days of prednisolone as first line therapy for acute GVHD identifies patients with different risk of transplant-related mortality and that second-line therapy with a combination of 6-methylprednisolone and anti-thymocyte globulin does not improve patient outcome compared to 6-methylprednisolone alone (Van Lint et al. 2006). Therefore, patients should be assigned to different risk groups as early as day +5 from the diagnosis of acute GVHD and hence could be eligible for different treatment strategies (Van Lint et al. 2006).

Chronic GVHD has a later onset than acute GVHD and has various clinical manifestations including: sclerodermatous skin lesions, kerato-conjuctivitis, sicca syndrome, lichenoid oral mucosal lesions, liver damage and pulmonary insufficiency (Sullivan et al. 1981). Despite immunosuppressive agents, approximately 30%–50% of patients will develop chronic GVHD after myeloblative HLA-identical sibling HSCT. However, the incidence of chronic GVHD may be higher after allogeneic HSCT using unmanipulated PBSCs (Champlin et al. 2000). Although limited chronic GVHD often resolves spontaneously with minimal intervention, extensive chronic GVHD requires prolonged immunosuppressive therapy and is associated with significant morbidity and mortality. More than 50% of patients with extensive chronic GVHD will ultimately die, mostly secondary to infections resulting from severe immune dysfunction (Atkinson et al. 1990).

The patient presented had an allogeneic HSCT for his ALL in the second CR. He initially developed mild acute GVHD of the skin which responded to steroid therapy, then he developed extensive chronic GVHD of skin, mouth and liver. Thereafter, the patient was admitted with acute exacerbation of his GVHD. Despite having GVHD of the lower GIT which could explain his symptomatology, the patient underwent several investigations including: repeated endoscopies with colonic biopsies, CMV antigenemia assays and stool cultures which revealed not only the severe form of lower GIT-GVHD, but also CMV disease of the colon as well as *Salmonella* colitis caused by food poisoning. The management of the patient was tailored according to his clinical condition and the laboratory findings and this included shifting him to ATG for better control of his GVHD and also shifting him to IV foscarnet for optimal control of his CMV colitis as there was evidence of clinical resistance to ganciclovir. The patient was supported with: appropriate antimicrobial cover; blood products as needed; GCSF and TPN to provide him with adequate nutritional requirements and to rest his severely inflamed bowel.

## Conclusion

Immunocompromised HSCT patients may develop severe complications at any time following their grafts and these complications are related to the pre-transplant conditioning regimens and to the immunosuppressive therapy administered to prevent graft rejection. Development of clinical and histological evidence of colitis should alert clinicians to take into account all the possible causes, and not to consider only one cause like GVHD, as other forms of colitis e.g. viral or bacterial can evolve either concomitantly or subsequently during the course of the illness. Appropriate screening tests including invasive ones and repeated cultures and serological investigations should guide the management in these high risk patients.
